# Validity study of the Turkish version of the Mini-Addenbrooke’s cognitive examination in mild cognitive impairment and Alzheimer’s disease

**DOI:** 10.1007/s13760-025-02983-w

**Published:** 2026-01-05

**Authors:** Cilem N. Evci, Ebru N. Barcin, Ali Ünal

**Affiliations:** 1 Turhal State Hospital, Tokat, Turkey; 2 Faculty of Medicine, Department of Neurology, Akdeniz University, Antalya, Turkey

**Keywords:** Mini-Addenbrooke’s cognitive examination, Cognitive test, Validity study, Alzheimer's disease, Mild cognitive impairment, Cognitive assessment screening tool

## Abstract

**Introduction & objective:**

The Mini-Addenbrooke’s Cognitive Examination (M-ACE) is an easy-to-administer and quick screening test that assesses four basic cognitive areas, namely orientation, verbal fluency, memory, and visuospatial abilities, out of 30 points. The objective of this study is to introduce M-ACE to the Turkish cognitive science literature and evaluate its applicability in Turkish society in comparison with the Mini-Mental State Examination (MMSE).

**Materials & methods:**

A total of 90 native Turkish speakers aged 60 and over with at least 12 years of formal education, including 30 patients with mild cognitive impairment (MCI group), 30 patients with Alzheimer’s disease (AD group), and 30 healty control subjects (control group), who applied to the neurology outpatient clinic between November 2020 and November 2021, were included in this prospective study. All participants were underwent MMSE and the Turkish version of M-ACE by experienced neurologists.

**Results:**

The optimal M-ACE cut-off values were 24.5 for differentiating MCI from controls (90% sensitivity, 73% specificity), 23.5 for distinguishing AD from controls (97% sensitivity, 90% specificity), and 19.5 for differentiating MCI from AD (70% sensitivity, 80% specificity).

**Conclusion:**

Turkish version of the M-ACE is a valid tool for assessing cognitive impairments in the elderly population.

## Introduction

Considering the limited time and resources in clinical practice, screening tests stand out as the first tool used for detailed neuropsychological assessment. Valid screening tests are patient-acceptable, readily administerable, largely independent of educational, cultural, and linguistic influences, and have high specificity and sensitivity. Screening tests remain crucial for guiding clinicians in differential diagnosis, identifying dementia in its early stages, and monitoring its course. The Alzheimer’s Association has listed 15 different cognitive screening tests for diagnosing dementia in primary care settings, but noted that none is a comprehensive assessment tool that can be used across all patient populations and settings. It has been recommended that specialized dementia clinics use screening tools that assess multiple cognitive domains, such as the Saint Louis University Mental Status (SLUMS) examination and the Montreal Cognitive Assessment (MoCA), whereas family physicians use those that can be administered in a shorter time and require less experience, such as the mini cognitive (Mini-Cog) test and the General Practitioner Assessment of Cognition (GPCOG) [1].

The Mini-Mental State Examination (MMSE) is the most frequently used screening test in Turkey, as well as worldwide, due to its ease of application in a short time [2]. However, given that the standardized MMSE requires competencies such as reading, writing, calculating, and forming sentences, difficulties arise in administering and subsequently evaluating individuals who are illiterate or have a low level of education. The limited educational opportunities in rural and socioeconomically disadvantaged areas, along with the low literacy rate, especially among older generations, have warranted the development of culturally adapted versions of the MMSE that do not require literacy. To this end, the MMSE has been adapted for use with illiterate individuals in China, India, several Latin American countries, and Turkey [3–6]. Accordingly, MMSE’s scope was modified to replace items specifically focused on reading and writing skills with items focused on visual and verbal cues. However, MMSE has a high false-negative rate in individuals with higher education levels and does not adequately assess executive functions and visuospatial abilities.

M-ACE was developed as a practical and time-efficient tool while maintaining high sensitivity in distinguishing the early stages of dementia. An original study evaluated the prognostic value of M-ACE total score cut-off values ​​<25 and < 21 for mild cognitive impairment (MCI) and dementia diagnoses, respectively (0.77 and 0.82, respectively), predicting mild cognitive impairment with adequate sensitivity (0.77) and specificity (0.82), and dementia with high sensitivity (0.92) but low specificity (0.61) [7]. M-ACE has higher sensitivity and is less likely to produce a ceiling effect compared to MMSE [7]. Additionally, M-ACE has been translated into many languages​, and its validity and reliability studies have already been conducted in different populations [8–10].

In view of the foregoing, this study’s objective is to validate the Turkish version of the M-ACE and evaluate its diagnostic accuracy (sensitivity and specificity) in patients with mild cognitive impairment and Alzheimer’s disease (AD) in comparison with the MMSE.

## Materials and methods

### Study design and setting

This prospective single-center study was conducted in the neurology outpatient clinic of Akdeniz University Hospital between November 2020 and November 2021. The study protocol was approved by the Clinical Research Ethics Committee of Akdeniz University Faculty of Medicine (Decision No: 70904504/666, Decision Date: 09/10/2020). The study was conducted in accordance with the ethical principles outlined in the Declaration of Helsinki. Written informed consent was obtained from all participants included in the study.

### Population and sample

The study population consisted of 118 consecutive patients aged 60 and above who presented with cognitive complaints. The inclusion criteria of the study were as follows: (a) having been diagnosed with AD or AD-related MCI according to the The National Institute on Aging—Alzheimer’s Association’s (NIA-AA) 2011 Diagnostic Criteria by an experienced neurologist for patients, (b) having scored 5/15 or lower on the Geriatric Depression Scale - Short Form (GDS-15), (c) being a native Turkish speaker, and (d) having received at least 12 years of formal education for both patients and control subjects. The study’s exclusion criteria for both patients and control subjects were as follows: (a) having been diagnosed with a neurological disease other than AD and MCI such as epilepsy, stroke, brain trauma or Parkinson’s disease, (b) having been diagnosed with other systemic diseases that may affect cognitive functions such as chronic renal failure, (c) having been diagnosed with a major psychiatric disorder or depression, (d) having been diagnosed with Alcohol Use Disorder according to the Diagnostic and Statistical Manual of Mental Disorders, Fifth Edition (DSM-5) diagnostic criteria, and (e) having a vision/hearing impairment that would impair test performance.

The control group consisted of healthy volunteers from among the patients’ relatives who had no cognitive complaints. In the end, a total of 90 participants, including 30 with MCI (MCI group), 30 with AD (AD group), and 30 control subjects (control group), were included in the study.

### Data collection

All participants underwent a routine neurological examination by a neurologist, as well as detailed laboratory tests, including vitamin B12 and vitamin D tests, liver, kidney, and thyroid function tests, and a complete blood count. Patients who had not undergone structural brain imaging via magnetic resonance imaging (MRI) or computed tomography (CT) within the previous six months underwent imaging tests before being included in the study.

All participants were also administered the Turkish versions of the Geriatric Depression Scale- Short Form (GDS-15), the Lawton and Brody Instrumental Activities of Daily Living (IADL), and MMSE, the validity and reliability studies of which have been previously completed. MMSE score of the AD group was below 26/30. MMSE scores of the MCI and control groups were above 26/30, and their IADL scores were unaffected but MCI group have progressive episodic memory complaints over the past six months. All cognitive screening tests were administered to the participants in a quiet, distraction-free environment by an experienced neurologist.

### M-ACE procedure

First of all, M-ACE was translated into Turkish in consultation with a team of experienced neurologists and clinical neuropsychologists, after obtaining the necessary permissions from the original research team that developed M-ACE. The name, surname, and address data used for the Turkish version of the Addenbrooke’s Cognitive Examination-Revised (ACE-R) were also used for M-ACE [11]. No additional adjustments were made to assess orientation, verbal fluency, and visuospatial abilities dimensions. The M-ACE is a screening test that can be administered in approximately 5 min. It is evaluated out of a total of 30 points, including prospective memory, verbal fluency, and memory dimensions out of 7 points each, visuospatial abilities (clock drawing) out of 5 points, and time orientation out of 4 points [7].

### Statistical analysis

SPSS 22.0 (Statistical Product and Service Solutions for Windows, Version 22.0, IBM Corp., Armonk, NY, U.S., 2013) software package was used to conduct the statistical analyses of the collected data. The results of the statistical analyses were expressed using descriptive statistics, i.e., mean ± standard deviation (SD) values for normally distributed continuous variables, median with interquartile ranges (IQRs) for non-normally distributed continuous variables, and frequencies (n) and percentage (%) values for categorical variables. The normal distribution characteristics of continuous variables such as age, years of education, and cognitive test scores were analyzed using the Shapiro-Wilk test and confirmed using histograms with Q-Q plots.

Intergroup comparisons of M-ACE and MMSE total scores across study groups were performed using the Kruskal-Wallis H test, as these data failed to meet the parametric assumptions. Post-hoc pairwise comparisons were performed with Dunn’s test with Bonferroni correction to control for Type I error rate inflation.

The correlations between M-ACE and MMSE scores were assessed using Spearman’s rank correlation coefficients (ρ), taking into account their robustness to nonlinear relationships and outliers. The agreement between M-ACE and MMSE was evaluated using Bland-Altman analysis, which involved calculating the mean deviation with 95% limits of agreement. Additionally, intraclass correlation coefficients (ICC) were calculated using two-way mixed effects models to evaluate their absolute agreement.

Diagnostic accuracy parameters were determined by receiver operating characteristic (ROC) curve analysis. Areas under the curve (AUCs) ​​were calculated using the DeLong test within 95% confidence intervals (CIs). Optimal cut-off values were determined by maximizing the Youden’s Index (J = sensitivity + specificity − 1) to balance sensitivity and specificity for clinical use. Positive and negative likelihood ratios were calculated to assess the diagnostic value of cognitive tests. ROC comparisons were validated using MedCalc Statistical Software version 19.2.6 (MedCalc Software Ltd, Ostend, Belgium).

Analyses stratified by gender were conducted to investigate potential gender-based differences in diagnostic thresholds. Cut-off values ​​for male and female subgroups were determined independently, where sample size permitted meaningful analysis. The Wilcoxon signed-rank test was used for paired comparisons between M-ACE and MMSE scores within each study group. All statistical tests were two-tailed. The significance threshold was set at α = 0.05.

## Results

There was no significant difference between the study groups in terms of age, gender, and education level (*p* = 0.73, *p* = 0.23, and *p* = 0.1, respectively). The mean total M-ACE score was 27.87 ± 1.11 (median-IQR = 28 − 1) in the control group, 21.87 ± 2.61 (median-IQR = 22 − 4) in the MCI group and 18.03 ± 3.22 (median-IQR = 18 − 5) in the AD group. Accordingly, the mean total M-ACE score was significantly lower in the AD group than in the MCI and control groups (*p* = 0.01). The mean total MMSE score was 29.37 ± 1 in the control group, 27.87 ± 1.11 in the MCI group and 24.10 ± 1.84 in the AD group. The total MMSE score was also the lowest, albeit not significantly, in the AD group compared to the MCI and control groups (*p* > 0.05).

In our study, both the M-ACE and MMSE demonstrated comparable diagnostic accuracy in distinguishing individuals with MCI from healthy controls, as reflected by identical Youden’s index values (0.633) and a non-significant difference in AUC (*p* = 0.495). Although the M-ACE showed slightly higher sensitivity, this difference was not statistically significant. Therefore, our findings suggest that both tests perform similarly in detecting mild cognitive impairment, in line with previous studies reporting comparable diagnostic efficiency between the two screening tools.

While the M-ACE total score cut-off value of 24.5 predicted MCI with 90% sensitivity and 73.33% specificity, the MMSE total score cut-off value of 28.5 predicted MCI with 76.67% sensitivity and 86.67% specificity. Both tools demonstrated excellent diagnostic performance in distinguishing between AD and control groups (0.969, 95% CI: 0.929–1.00.929.00 for M-ACE vs. 0.996, 95% CI: 0.986–1.00.986.00 for MMSE; *p* < 0.001). The MMSE demonstrated significantly higher discriminative power than the M-ACE in distinguishing MCI from AD patients (AUC = 0.987 vs. 0.821, *p* = 0.002). (Table 1).

**Table 1:** Diagnostic performance comparison of M-ACE and MMSE

Diagnostic Parameters	Control-MCI		Control-AD		MCI-AD	
	MMSE	M-ACE	MMSE	M-ACE	MMSE	M-ACE
**AUC** *(95% CI)*	0.839 (0.735–0.944)	0.875 (0.783–0.967)	0.996 (0.986–1.00.986.00)	0.969 (0.929–1.00.929.00)	0.987 (0.970–1.00.970.00)	0.821 (0.716–0.925)
**p-value**	< 0.001	< 0.001	**< 0.001**	**< 0.001**	**< 0.001**	**< 0.001**
**Cut-off Point**	28.5	24.5	26.5	23.5	26.5	19.5
**Sensitivity** *(%)*	76.67	90	100	96.67	100	70
**Specificity** *(%)*	86.67	73.33	96.67	90	90	80
**Positive Likelihood Ratio**	5.75	3.38	30	96.67	10	3.5
**Negative Likelihood Ratio**	0.27	0.14	0	0.04	0	0.38
**Positive Predictive Value** *(%)*	85.19	77.14	96.77	90.62	90.91	77.78
**Negative Predictive Value** *(%)*	78.79	88	100	96.43	100	72.73
**Accuracy** *(%)*	81.67	81.67	98.33	93.33	95	75
**Youden’s Index**	0.633	0.633	0.967	0.867	0.9	0.5
**AUC Difference**	Reference	0.036 (*p* = 0.495)	Reference	−0.027 (*p* = 0.107)	Reference	**−0.166 (***p*** = 0.002)**

Bold p-values indicate statistical significance (*p* ≤ 0.05)

M-ACE: Mini-Addenbrooke’s Cognitive Examination; MMSE: Mini Mental State Examination; MCI: Mild Cognitive Impairment; AD: Alzheimer’s Disease; AUC: Area Under the Curve; CI: Confidence Interval

M-ACE orientation, verbal fluency, and visuospatial abilities (clock drawing) scores were able to distinguish the AD group from the control and MCI groups (*p* = 0.001, *p* = 0.01, and *p* = 0.01, respectively), but could not distinguish between the control and MCI groups. M-ACE memory and recall scores of the control group were significantly higher than those of the MCI and AD groups (*p* = 0.01 for both cases), with no significant difference between the MCI and AD groups. While there were no significant differences between the groups in MMSE orientation and registration memory scores, other scores (attention, calculation, recall, language, and visuospatial abilities) distinguished the AD group from the MCI and control groups (*p* = 0.01).

Agreement and correlation analyses between M-ACE and MMSE (Table 2) revealed significant differences in all groups (Wilcoxon signed-rank test, *p* < 0.001 for all cases). In the Bland-Altman analysis, the mean deviation was calculated as −3.667 in the control group, −6.000 in the MCI group, and − 6.067 in the AD group.

**Table 2:** Agreement and correlation analyses between M-ACE and MMSE

Group	Wilcoxon Signed-Rank Test		Bland-Altman Analysis			Agreement Coefficient			Correlation	
	z	*p*	Mean Bias	Lower Limit	Upper Limit	ICC	95% CI	*p*	*r*	*p*
**Control**	0	< 0.001	−3.667	−7.495	0.162	0.427	0.085–0.679	0.178	0.348	< 0.001*
**MCI**	0	**< 0.001**	−6.000	−6.980	−5.024	0.150	−0.217-0.479	0.008	**0.473**	**< 0.001**
**AD**	0	**< 0.001**	−6.067	−7.120	−5.020	0.425	0.082–0.678	0.003	**0.524**	**< 0.001**

Bold values indicate statistical significance (*p* ≤ 0.05). *Wilcoxon test results: Control (t = −10, df = 29), MCI (t = −13, df = 29), AD (t = −12, df = 29), all groups *p* < 0.001 **Spearman correlation coefficients

MCI: Mild Cognitive Impairment; AD: Alzheimer’s Disease; ICC: Intraclass Correlation Coefficient; CI: Confidence Interval; r: Spearman correlation coefficient

ICCs (0.427 for the control group, 0.150 for the MCI group, and 0.425 for the AD group) indicated a low level of agreement between M-ACE and MMSE. While there was a weak positive correlation between MMSE and M-ACE total scores in the control group (*r* = 0.348, *p* < 0.001), a moderate positive correlation was found in the MCI (*r* = 0.473, *p* < 0.001) and AD (*r* = 0.524, *p* < 0.001) groups.

Analysis of the predictive powers of gender-specific M-ACE total score cut-off values ​​revealed that a cut-off value of 22.5 predicted MCI with 81% diagnostic accuracy in males and a cut-off value of 23.5 M predicted MCI with 79% diagnostic accuracy in females (*p* = 0.01), while a cut-off value of 19.5 predicted AD with 95% diagnostic accuracy in males and a cut-off value of 18.5 predicted AD with 89% diagnostic accuracy in females (*p* < 0.05). On the other hand, the predictive powers of gender-specific M-ACE cut-off values ​​could not be analyzed due to an insufficient number of females in the control group.

## Discussion

The study’s primary findings were that there was a positive correlation between the total score of the Turkish version of M-ACE and the total score of MMSE. The optimal total M-ACE cut-off scores for AD and MCI diagnoses were 23.5/30 and 24.5/30, respectively. The cut-off score determined for diagnosing AD showed higher diagnostic power than that for MCI (sensitivity = 96.7%, specificity = 90%, AUC = 0.969, J = 0.867 vs. sensitivity = 90%, specificity = 73%, AUC = 0.875, J = 0.633). By comparison, the corresponding MMSE cut-off scores were 26.5/30 for AD (sensitivity = 100%, specificity = 96.7%, AUC = 0.996, J = 0.967) and 28.5/30 for MCI (sensitivity = 76.7%, specificity = 86.7%, AUC = 0.839, J = 0.633).

The original validity and reliability study of the M-ACE screening test reported that a M-ACE total score cut-off value of ≤ 25/30 predicted dementia with high sensitivity and specificity, and that M-ACE was more sensitive and showed less ceiling effect than MMSE [7]. Sensitivity and specificity analyses indicate that all M-ACE total score cut-off values have higher sensitivity but slightly lower specificity than those of MMSE in identifying individuals with cognitive impairments and that M-ACE is helpful in assessing and monitoring patients in the clinical setting, especially those with MCI [7, 8, 12–14]. In comparison, we found that M-ACE total score predicted AD diagnosis with a sensitivity similar to those reported in the literature, yet with lower specificity. In contrast to their comparable performance in distinguishing MCI from healthy controls, the MMSE demonstrated significantly higher discriminative power than the M-ACE in differentiating MCI from AD patients (AUC = 0.987 vs. 0.821, *p* = 0.002). This finding suggests that the MMSE may be more effective in identifying the transition from mild cognitive impairment to Alzheimer’s disease, likely due to its stronger sensitivity to global cognitive decline. Nevertheless, the M-ACE retains clinical value, particularly for detecting early and subtle cognitive changes that may not yet meet the threshold for dementia.

M-ACE orientation, verbal fluency, and visuospatial abilities scores were able to distinguish between the AD and MCI groups, while the M-ACE memory and recall scores distinguished the control group from both the MCI and AD groups. The lower M-ACE memory and recall scores of AD and AD-related MCI patients compared to those of control subjects reflect episodic memory impairment in the early stages of AD and AD-related MCI. While the MMSE primarily assesses language functions, it is less discriminatory in clinical practice because it cannot adequately assess executive functions and semantic-visual memory [15].

M-ACE provides a more detailed assessment of the cognitive profile compared to the MMSE. M-ACE offers several advantages, including being easily translatable from English to other languages, not requiring administration by an expert, and being completed in approximately the same time as the MMSE [15, 16]. However, as indicated by the positive correlation between the M-ACE total score and education level, the M-ACE total score is affected by the education level of the individual to whom it is applied [8, 16]. Therefore, a lower M-ACE total score cut-off value of 16.5 has been used to distinguish patients with AD from control subjects in some populations (it is recommended to increase the M-ACE total score cut-off value to 17.5 to increase its sensitivity) [17]. Since we only included individuals with 12 years or more of education in our study, we were unable to evaluate the correlation between the M-ACE total score and education level.

### Limitations

This study’s primary limitation is that only patients with AD and AD-related MCI were included, while patients with other diagnoses, such as frontotemporal dementia and vascular dementia, were excluded, precluding any conclusions about the diagnostic discriminatory power of the M-ACE. Secondly, the inability to determine a M-ACE total score cut-off value for individuals with lower levels of education, as only those with 12 or more years of education were included, could be considered another limitation of the study. A new study could be planned with a larger number of participants diagnosed with different neurodegenerative dementias, including those with lower levels of education, to obtain more detailed data. Thirdly, in this study, the classification of participants was based on MMSE performance adjusted for higher education levels. Although Güngen et al. reported a dementia cut-off of 23/24 for the Turkish population, we applied a higher threshold because all participants had at least 12 years of education. Consequently, individuals with MMSE scores below 26 were classified as having early-stage Alzheimer’s disease. This approach may have influenced group distribution but was necessary to avoid underestimating cognitive impairment in highly educated individuals.

## Conclusion

The Turkish version of the M-ACE demonstrated good diagnostic accuracy in differentiating MCI and AD from healthy controls, with optimal cut-off values of 24.5 for distinguishing MCI from controls and 23.5 for distinguishing AD from controls. Therefore, it can be considered a reliable and practical screening tool for identifying cognitive impairment in clinical settings.

## Data Availability

Data are available from the corresponding author upon reasonable request, if deemed necessary by the reviewers.

## Abbreviations

AD: Alzheimer’s Disease
AUC: Area Under the Curve
AUD: Alcohol Use Disorder
CT: Computed Tomography
DSM: 5—Diagnosticand Statistical Manual of Mental Disorders, 5th Edition
GDS: Geriatric Depression Scale
GPCOG: General Practitioner Assessment of Cognition
IADL: Instrumental Activities of Daily Living
IQR: Interquartile Range
M: ACE—Mini—Addenbrooke’s Cognitive Examination
MCI: Mild Cognitive Impairment
MMSE: Mini Mental State Examination
MOCA: Montreal Cognitive Assessment
MRI: Magnetic Resonance Imaging
NIA: AA—National Institute on Aging—Alzheimer’s Association
ROC: Receiver Operating Characteristic
SD: Standard Deviation
SLUMS: Saint Louis University Mental Status Examination
